# Cryogenic fracturing using liquid nitrogen on granite at elevated temperatures: a case study for enhanced geothermal systems in Kazakhstan

**DOI:** 10.1038/s41598-023-50223-z

**Published:** 2024-01-02

**Authors:** Sotirios Nik. Longinos, Randy Hazlett

**Affiliations:** Department of Petroleum Engineering, School of Mining and Geosciences, Nazarbayev University, Astana, Kazakhstan

**Keywords:** Energy science and technology, Engineering

## Abstract

Cryogenic fracturing using liquid nitrogen (LN_2_) is a novel stimulation technology that enhances porosity, permeability, and rock-fluid contact area in subsurface formations targetted for geothermal energy extraction. In our experimental study, granite cores collected from the Zhylgyz region in South Kazakhstan were equilibrated at various elevated temperatures before treatments involving LN_2_ exposure time. Compression, Brazilian, and fracture toughness tests were performed on granite with starting temperatures ranging from 100 to 500 °C to quantify the impact of initial temperature on cryogenic fracturing and to compare with baseline geomechanical tests at 50 °C without LN_2_ exposure. The results show that LN_2_ cooling of hot granite induces mechanical rock failure and permeability enhancement. Moreover, the degree of thermo-fracturing augments with initial granite temperature, total freezing time, and number of freezing–thawing cycles. The peak load before failure of granite specimens, both in compression and Brazilian tests, reduces with the increased sample temperature difference and length of LN_2_ treatment. The fracture toughness of our semi-circular bend (SCB) LN_2_-treated specimens diminished with increasing temperature difference between granite and boiling point. In both experimental LN_2_ treatment processes, the specimens with an initial temperature of 500 °C before LN_2_ treatment formed many new fissures and extensions of pre-existing ones, showing that the plastic behavior is augmented. While cryo-fracturing experimental confirmation is recommended with site-specific samples in planning geothermal operations, these results in our work indicate a threshold downhole temperature, e.g., > 300 °C, for enhanced stimulation outcomes.

## Introduction

The energy transition from fossil fuels to environmentally more friendly energy resources is paramount to today’s civilization^1–3^. The main objective is advancing and implementing renewable energy resources^4–6^. Geothermal energy, with low carbon emission and abundant resources, is one of the energy transition pillars^7–10^. Hot dry rock (HDR) or enhanced (engineered) geothermal systems (EGS) is a technology for developing deep geothermal resources that store abundant thermal energy^11–13^ hosted in low-permeability rocks (0.001 to 0.1 md^14,15^) such as granite, gneiss, or granodiorite. Hence, stimulation methods are essential to create a fracture network to increase thermal heat transfer capacity^16,17^. Commonly employed stimulation methods include conventional hydraulic fracturing, thermal fracturing, shear fracturing, chemical stimulation, acidizing, and multilateral wells^15,18–22^. EGS by hydraulic fracturing (mainly multi-zone) commonly outperforms other methods^18,23,24^. However, hydraulic fracturing creates concerns over extensive water use, formation damage, contingent surface or groundwater contamination, and the cost of chemical additives^25–27^.

Waterless fracturing provides an alternative. Waterless fracturing could contingently comprise liquid/supercritical carbon dioxide (LCO_2_/SC-CO_2_), liquified natural gases (LNG), liquified petroleum gases (LPG), and liquid nitrogen (LN_2_)^13,28,29^. The LN_2_ boiling point at atmospheric conditions is −196 °C, providing the highest temperature difference between reservoir formation and injecting fluid (up to 300 °C^30^), which enhances thermal fracturing potential. Additional benefits of LN_2_ include that it is inodorous, inert (non-reactive), and not harmful to humans or the environment. Due to the above benefits, LN_2_ was effectively employed for coalbed methane (CBM) production in the 1990s^31,32^. On the other hand, there was reasonable concern about the transfer of proppants to preserve permeability gains during stimulation. A potential solution could involve a higher injection fluid flow rate and ultra-lightweight proppants^33,34^.

Recently, many research groups, mainly from China, examined the effect of LN_2_ cooling in granite rocks in laboratory conditions. Zhang et al. examined granite samples subjected to a low-pressure liquid nitrogen jet. These researchers concluded that the heating process has a small influence on thermal-crevice formation compared to LN_2_ cooling^35^. Yang et al. investigated granite samples under triaxial-confining stresses, comparing base case rock strength with that following LN_2_ cooling. They determined that LN_2_ cryogenic stimulation could diminish the breakdown pressure level by 9–51% more than the untreated samples^14^. Wu et al. investigated the influence of the freezing–thawing process with LN_2_ treatment on granite's physical and mechanical properties. Their outcomes defined that the heating and LN_2_ treatment process can impair the granite structural integrity, enhancing permeability. Moreover, they also indicated that granites with smaller particle sizes are more sensitive to the heating and LN_2_- treatment cycle^36^.

Zhou et al. compared heated granite specimens with LN_2_ and natural cooling. They determined that LN_2_ treatment urges more significant initial thermal failure, leads to greater failure before peak stress, and makes granite failure easier than natural cooling^37^. Su et al. examined marble specimens with LN_2_ treatment. The outcomes showed that the porosity of samples augmented significantly after LN_2_ treatment, whereas the P-wave velocity, compression strength, and elastic modulus were reduced^38^. Li et al. examined elevated heated granites with LN_2_ cooling under uniaxial compression and acoustic emission (AE) monitoring tests. They showed that with cycle augmentation, AE ring counts and amplitude of energy increase, while more precisely, when the granite reaches the damage stage, the energy at the failure point is enhanced, and the ring count also augments sharply^39^. Lai et al. examined heated granites with LN_2_ treatment through nuclear magnetic resonance (NMR) and ultrasonic tests. Their outcomes indicated that with the augmentation of heating temperature and the number of cold shocks, the porosity of the specimens was enhanced. The wave velocity diminishes, and the time delay and deformation of the waveforms become more prominent^40^. Qu et al. investigated the mechanical properties of granite specimens of heated samples after LN_2_ treatment. The sample failure predominantly occurred at the initial LN_2_ cooling stage in the core’s outer region. They found that the more significant the temperature difference, the more the LN_2_ treatment influences granite failure. Moreover, the granite mineral content significantly affected the mechanical strength. A decrease in quartz content reduced mechanical strength^41^.

This paper examines the effect of cryogenic treatment options to assess a possible waterless stimulation method in different granite samples from the southern region of Kazakhstan. It should be mentioned that Kazakhstan is one of the driest nations in the world, ranking 160^th^ among 186 surveyed countries; hence, the use of millions of gallons of water for stimulation processes is problematic. Our examination includes the systematic investigation of the mechanical properties of granite specimens from the Zhylgyz region in South Kazakhstan by three different unconfined experimental methods: uniaxial compression stress (UCS), Brazilian test, and fracture toughness mode I test. The granite samples were initially placed in a drying oven to eliminate any possible moisture before being placed in a muffle furnace at elevated temperature levels. This was followed by LN_2_ treatment with two processes: freezing time and multiple freezing–thawing cycles.

## Experimental process and equipment

**Figure 1:**
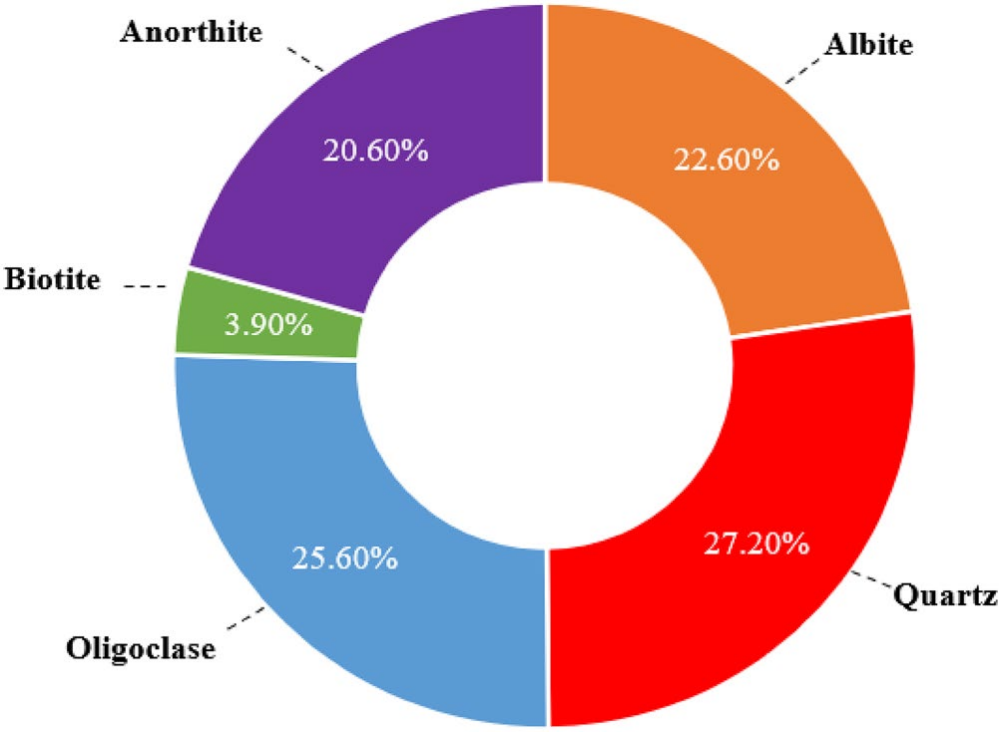
X-ray diffraction (XRD) composition for sampled Kazakhstani granite.

Granite specimens were selected and collected from outcrops from the Zhylgyz region of southern Kazakhstan. X-ray diffraction (XRD) is a method used to identify the structural characteristics of crystalline rock, powder, or other material. The pie chart (Fig. 1) shows the composition of the minerals in the granite structure. Albite is the most abundant mineral present in the sample, with 27.2% of the total composition. Other minerals such as quartz, oligoclase, biotite, and anorthite constitute 25.6%, 20.6%, 3.9%, and 22.6% of the granite sample.

**Figure 2:**
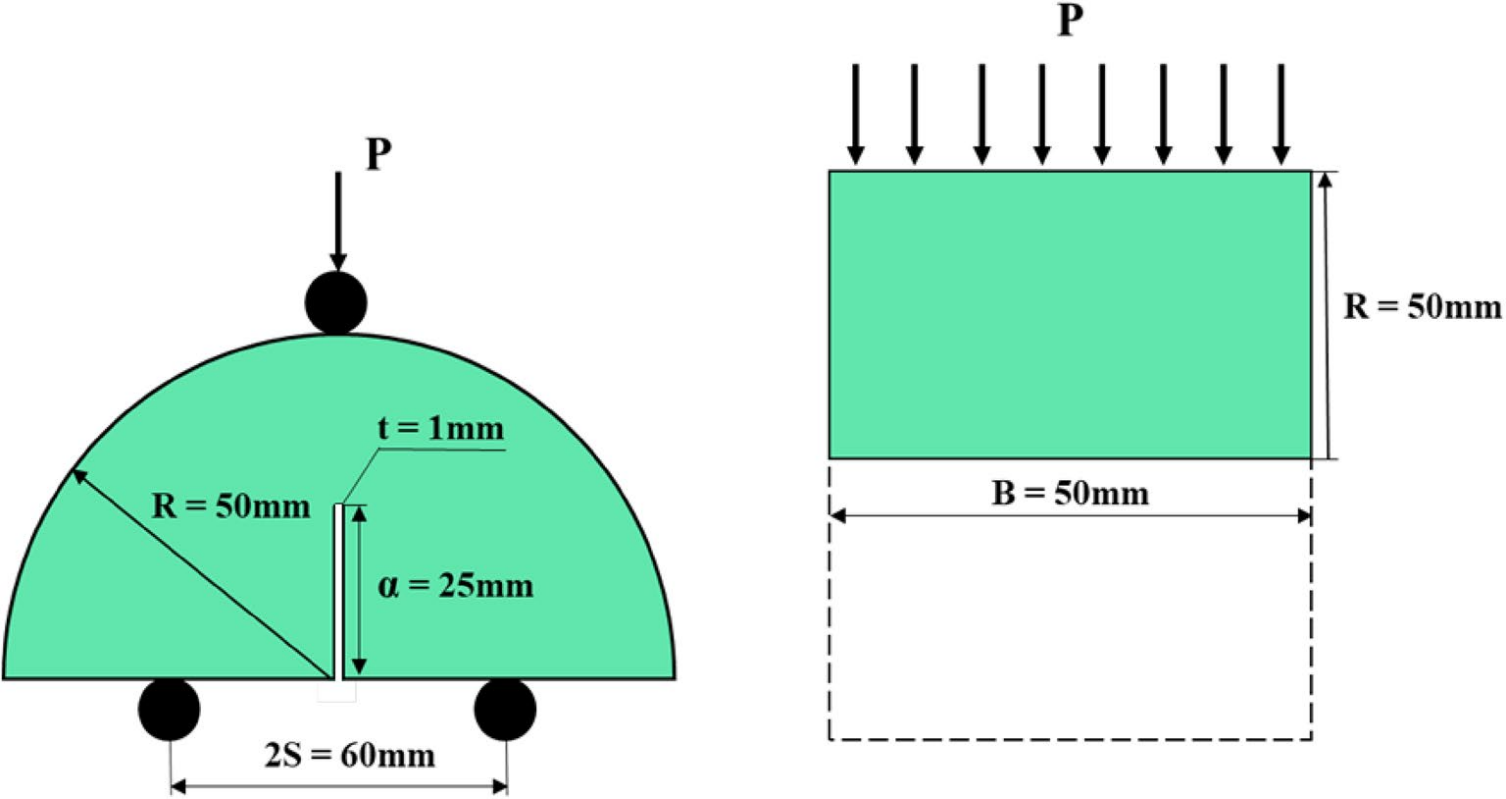
Semi-circular bend granite geometry.

Specimens for compression and ultrasonic tests were 50 mm in diameter and 100 mm in length. For permeability tests, specimens were 32 mm in diameter and 50 mm in length. For Brazilian tests (indirect tensile stress), samples were 50 mm in diameter and 25 mm in length. For the fracture toughness test, a basic geometric model of a semi-circular bend (SCB) sample is presented in Fig. 2. P is the force (load) applied at the top of the granite specimen, B is the sample thickness and crack length, R is the sample radius, and 2S is the distance between the two bottom support rollers. The geometrical dimensions of the SCB sample in this research work and the ISRM-recommended values are presented in Table 1.

**Table 1:** Dimensions of granite SCB specimens.

Dimensions	Dimensions in present research work according to ISRM recommended values
Diameter (D)	100 mm (larger than 109-grain size or 76 mm)
Thickness (B)	50 mm (larger than 4D or 30 mm)
Crack length (α_ο_)	*α/R* = 0.5 (0.4 ≤ *α* ≤ 0.6)
Span length (S)	*S/2R* = 0.6 (0.5 ≤ *S/2R* ≤ 0.8)

**Figure 3:**
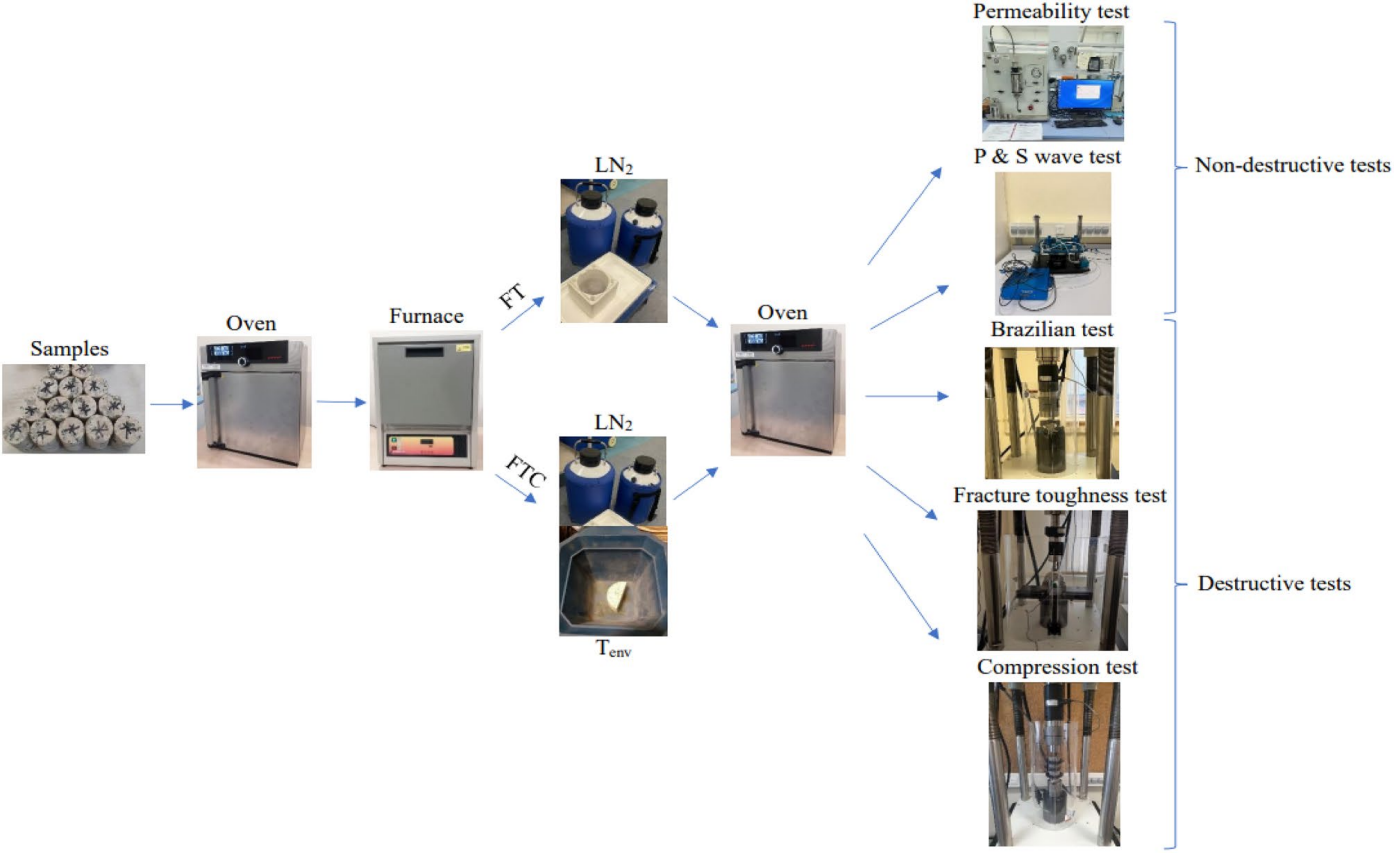
Experimental process for FT and FTC methods.

Mode I fracture toughness K_IC_ can be defined by the maximum force (load) P_*max*_, the dimensions of the SCB sample, and the non-dimensional parameter Y_I_; the formula is as follows^35^:

1 $$ K_{IC} = \, P_{max} \times \, (\pi \times\alpha )^{1/2} \times \, Y_{I} $$

2 $$ Y_{I} = \, - \, 1.297 \, + \, 9.516\left( {S/2R} \right) \, - \, \left( {0.47 \, + \, 16.457\left( {S/2R} \right)} \right) \, \beta \, + \, \left( {1.071 \, + \, 34.401\left( {S/2R} \right)} \right) \, \beta^{2} $$

3 $$ \beta \, = \alpha /R $$

where P*max* is the maximum load (kN), which can be obtained directly from the load-time curve; Y_I_ is a non-dimensional stress intensity factor determined by the dimensions of the SCB sample. The average grain size in our granite samples was less than 5 mm. Figure 3 shows the different equipment used in our experiments.

Specimens were pre-conditioned in a drying oven for 24 h at 50 °C. Analysis was performed using two different experimental methods: (i) a freezing time (FT) process, where specimens were placed in a furnace and heated at a rate of 10 °C/min and held for two hours at final temperatures of 100 °C, 300 °C, or 500 °C to span the practical range of downhole temperatures of geothermal prospects in Kazakhstan and to compare results to existing literature^42,43^. Samples were then immersed in LN_2_ for 1 h; (ii) a freezing–thawing cycle (FTC) process, where specimens were submerged in LN_2_ for 5 min after two hours of heating and then subjected to room temperature conditions for 5 min, completing one cycle. The freezing–thawing cycle procedures were repeated for 12 cycles (C12). At the end, the samples were placed at 50 °C in the drying oven for 2 h before their examination. Figure 3 shows the flow chart of the experimental process with FT and FTC methods. Process experiments, permeability measurements, and acoustic experiments were duplicated to assess repeatability and accuracy.

## Results of mechanical and physical parameters

### Uniaxial compression tests

**Figure 4:**
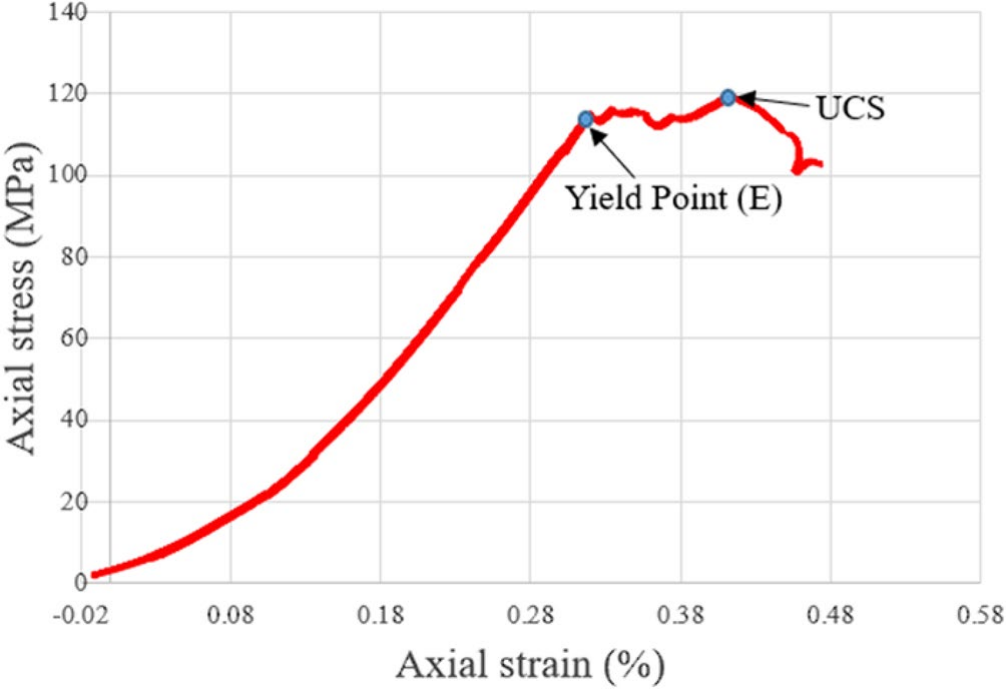
Typical axial stress versus axial strain with deformation in the uniaxial compression tests on granites with elevated initial temperatures with LN_2_ treatment.

**Figure 5:**
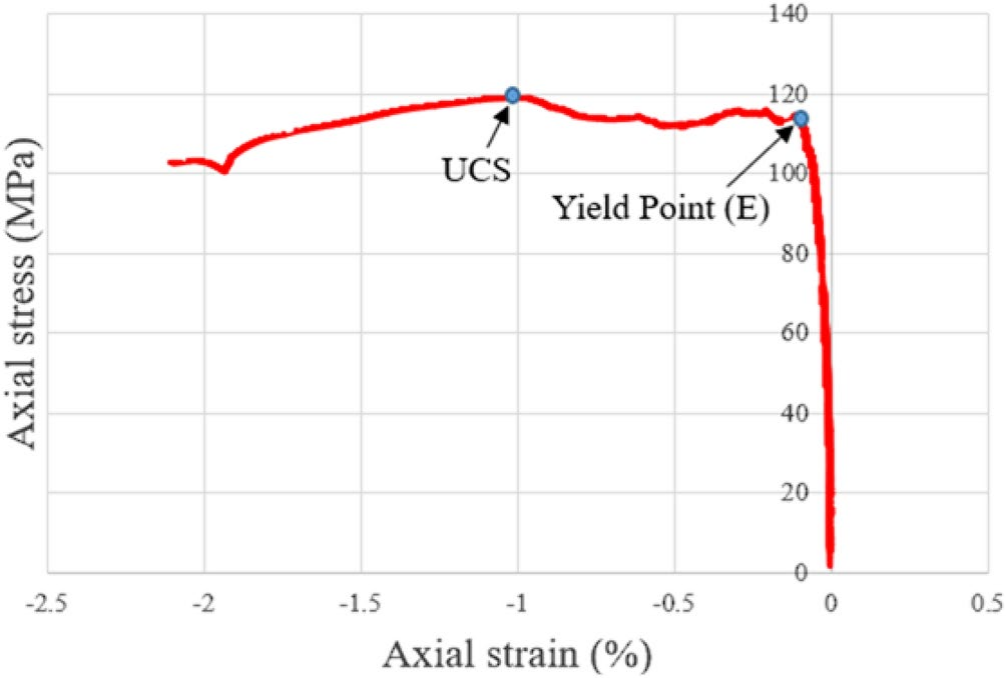
Typical axial stress versus radial strain with deformation in uniaxial compression tests on granites deformed with elevated initial temperatures with LN_2_ treatment.

For all the tested granites with elevated initial temperatures before LN_2_ treatment, the mechanical results of the uniaxial compression strength (UCS) experiments followed the typical five stages of deformation. Primarily, the axial stress–axial strain curves were nonlinear because of the closure of the micro-crevices oriented sub perpendicular to the compression direction and the sample-loading interfacial deformation, where almost zero radial strain exists. Then, the specimens indicated a linear augmentation in axial stress and axial and radial strain. This area finished at the onset of the yield point denoted E (Figs. 4 and 5), marked by a deviation from the linearity of the axial stress versus axial strain curves. After attaining E, the axial stress versus strain curves followed a non-linear augmentation until the maximum axial stress (curve peak). Finally, the specimen went into the stress failure phase and macroscopically collapsed.

**Figure 6:**
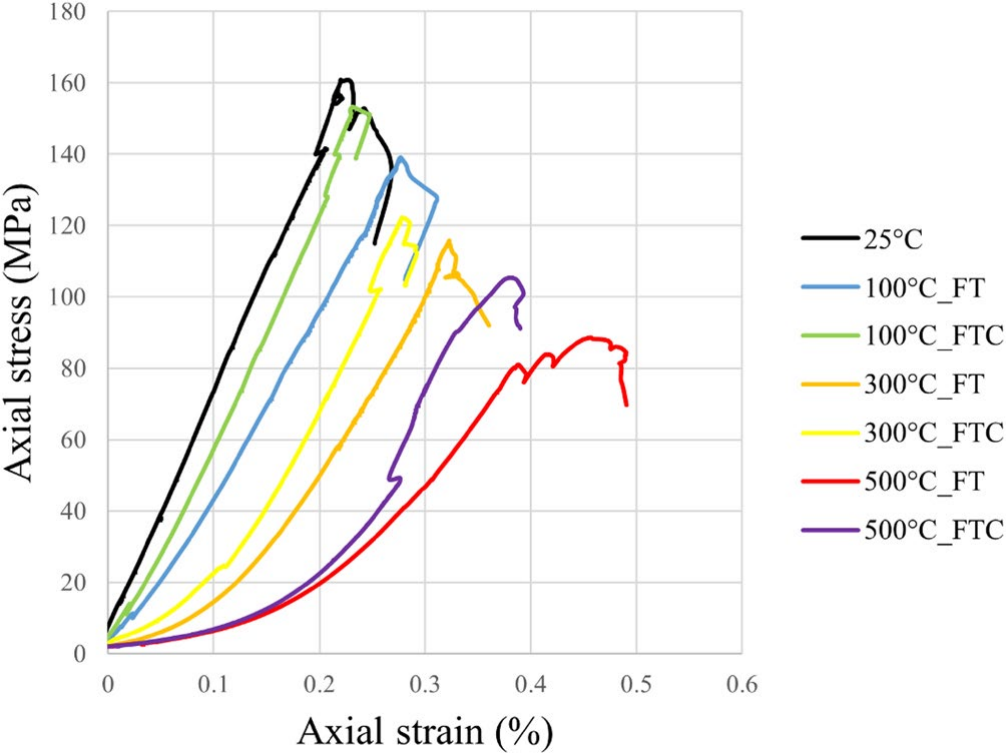
Axial stress versus axial strain test for both freezing time and freezing–thawing cycle processes in UCS testing for initial experiments.

**Figure 7:**
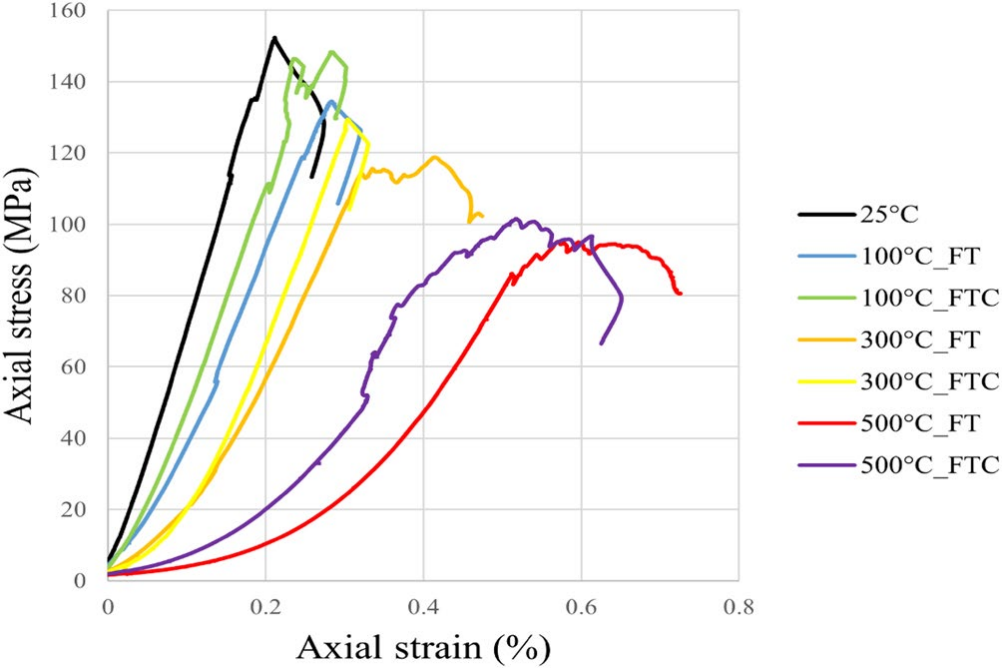
Axial stress versus axial strain retest for both freezing time and freezing–thawing cycle processes in UCS testing for repeated experiments.

**Figure 8:**
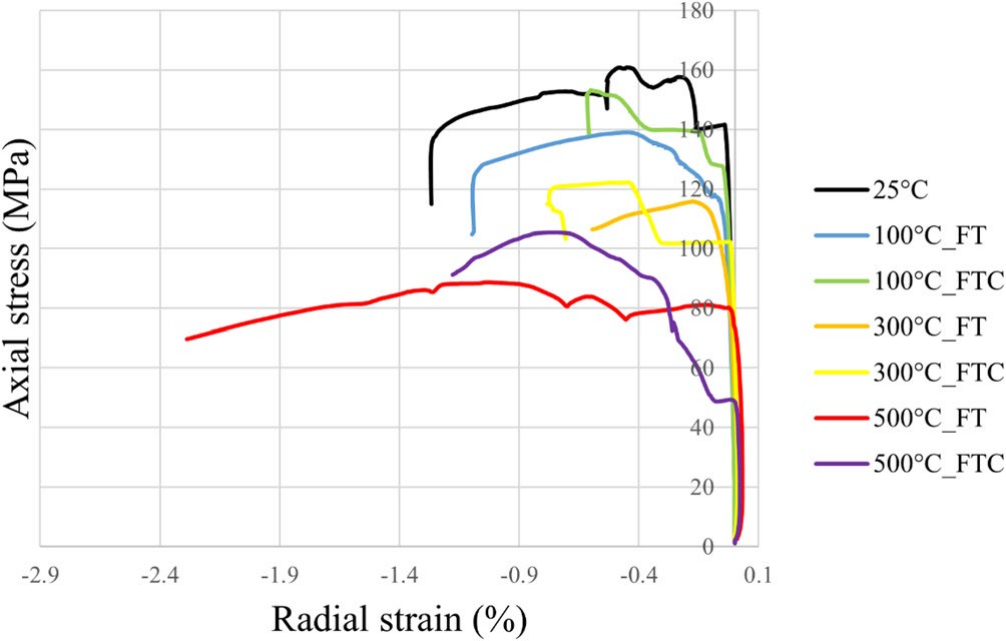
Axial stress versus radial strain for both freezing time and freezing–thawing cycle processes in UCS testing for initial experiments.

**Figure 9:**
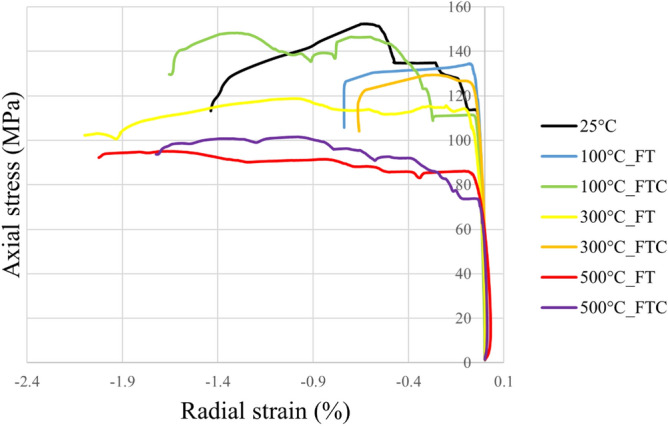
Axial stress versus radial strain retest for both freezing time and freezing–thawing cycle processes in UCS testing for repeated experiments.

**Figure 10:**
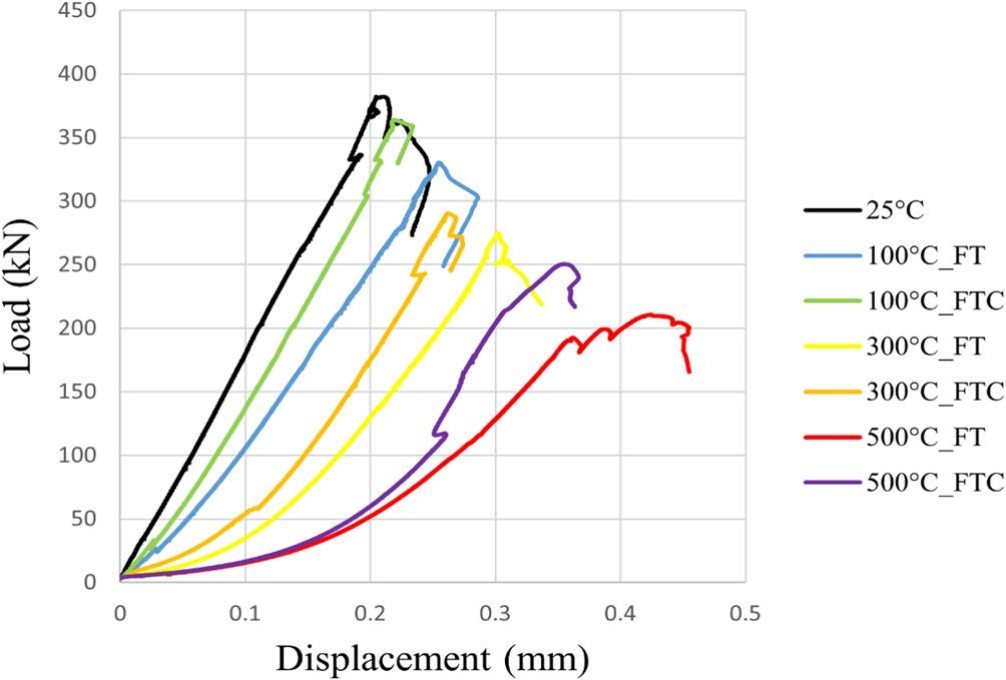
Load versus displacement for both freezing time and freezing–thawing cycle processes in UCS testing for initial experiments.

**Figure 11:**
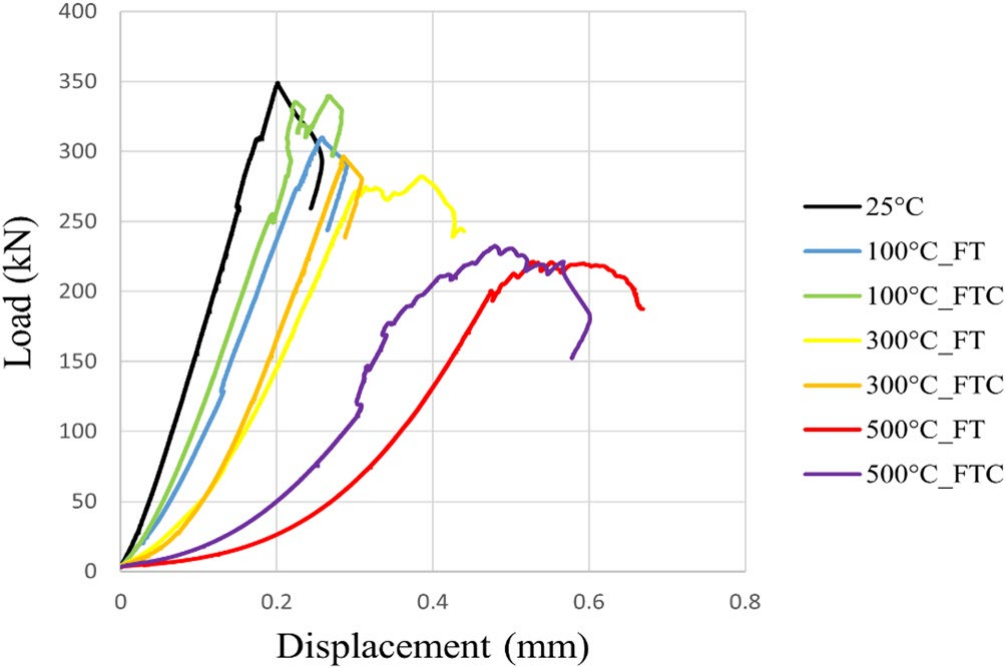
Load versus displacement retest for both freezing time and freezing–thawing cycle processes in UCS testing for repeated experiments.

UCS tests were performed to examine the strength of treated granite specimens. The strength and deformation outcomes were obtained by estimating axial stress versus axial strain, axial stress versus radial strain, and load versus displacement figures for the freezing and freezing–thawing cycle cryogenic treatment methods. Figures 6 and 7 present the axial stress versus axial strain curves. Figures 8 and 9 show axial stress versus radial strain curves for the two different LN_2_ treatment processes, and Figs. 10 and 11 indicate load versus displacement for the same treatment processes.

The maximum measured stress in the experiment with no treatment was 156.6 MPa (average value, Table 2). The lowest values occur at 500 °C with LN_2_ treatment with 91.9 MPa and 103.5 MPa for FT and FTC experiments. The stress of specimens with temperatures (until 300 °C) after LN_2_ cooling is high with minor alterations among each other, which varies for difference per experiment (percentage difference in USC peak stresses between successive temperature increments) from 11.7% (difference between no treatment and 100 °C with LN_2_ treatment experiments) to 45.7% (difference between no treatment and 500 °C with LN_2_ treatment experiments) for FT experiments and 2.6% to 26.5% for FTC, respectively. On the other side, the difference per temperature (percentage difference in USC peak stresses between the baseline and elevated temperature increments) varies from 11.7% (difference between no treatment versus 100 °C and LN_2_) to 44.9% (difference between no treatment versus 500 °C and LN_2_) for FT experiments and from 2.6% to 34.5% for FTC experiments respectively. The last column of Table 2 shows the standard error. The standard error in measured UCS peak stress varies by 1.5% to 4.3%, confirming the consistency of our research work. The difference between reported experiments is the percentage difference in USC peak stress between similar tests with successive differences in initial temperature, while the difference per temperature shock is the percentage difference in USC peak loads between the baseline and elevated temperature increments for both FT and FTC experiments.

**Table 2:** Results from UCS test experiments for stress values for both FT and FTC experiments.

Specimen number	Temperature (°C)	UCS peaks (kN), FT/FTC	Difference per experiment (%), FT/FTC	Difference per temperature (%), FT/FTC	Average value (kN), FT/FTC	Standard error (%), FT/FTC
1	25	160.9	–	–	156.6	4.3
2		152.4				
1	100	139.1/153.3	−13.6/−4.7	−13.6/−4.7	136.8/150.8	2.3/2.5
2		134.5/148.4	−11.7/−2.6	−11.7/−2.6		
1	300	115.8/122.2	−16.7/−20.3	−28.0/−24.0	117.3/125.8	1.5/3.6
2		118.8/129.4	−11.8/−12.8	−22.0/−15.0		
1	500	88.6/105.4	−45.7/−26.5	−44.9/−34.5	91.9/103.5	3.2/1.9
2		95.1/101.6	−20.0/−21.5	−37.6/−33.3		

**Figure 12:**
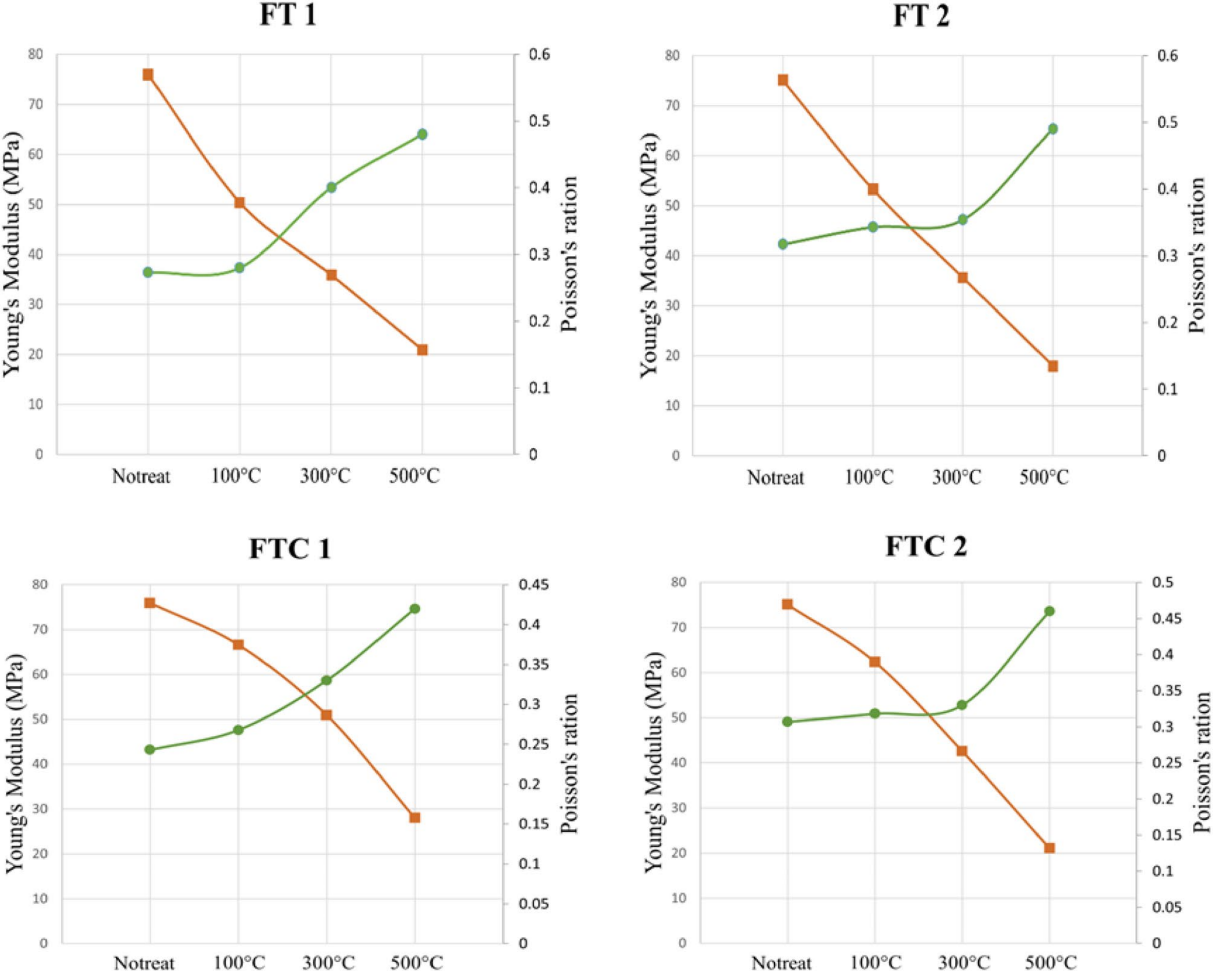
Young modulus and Poisson’s ratio values for FT experiments (upper row) initial (left) and repeated (right) and FTC experiments (lower raw) initial (left) and repeated (right).

Poisson’s ratio and Young modulus are important mechanical characteristics calculated in the UCS tests. Alterations in Poisson’s ratio and Young modulus must be defined to accurately predict fracture initiation and propagation. Figure 12 shows the relationship between the Young modulus and Poisson's ratio with elevated temperatures with LN_2_ treatment. As initial temperature increases along with LN_2_ treatment, Young modulus decreases. When granite specimens were heated to 500 °C and treated with LN_2_, the values of Young modulus decreased by 72.5% and 76.15% for FT experiments and 63.11% and 70.1% for FTC experiments compared to the baseline experiment. Concerning Poisson’s ratio, the opposite trend is observed, as Poisson’s ratio increased as the range of the temperature shock increased. When granite samples were heated at 500 °C and treated with LN_2_, the values of the Poisson’s ratio augmented by 43.1% and 35.5% for FT experiments and 35.0% and 31.1% for FTC experiments compared to the baseline experiment. The initial slight augmentation of Poisson’s ratio may have occurred because specimens pre-equilibrated up to 100 °C become dry, increasing their elasticity. This observation is consistent with the work of other researchers^44,45^.

**Figure 13:**
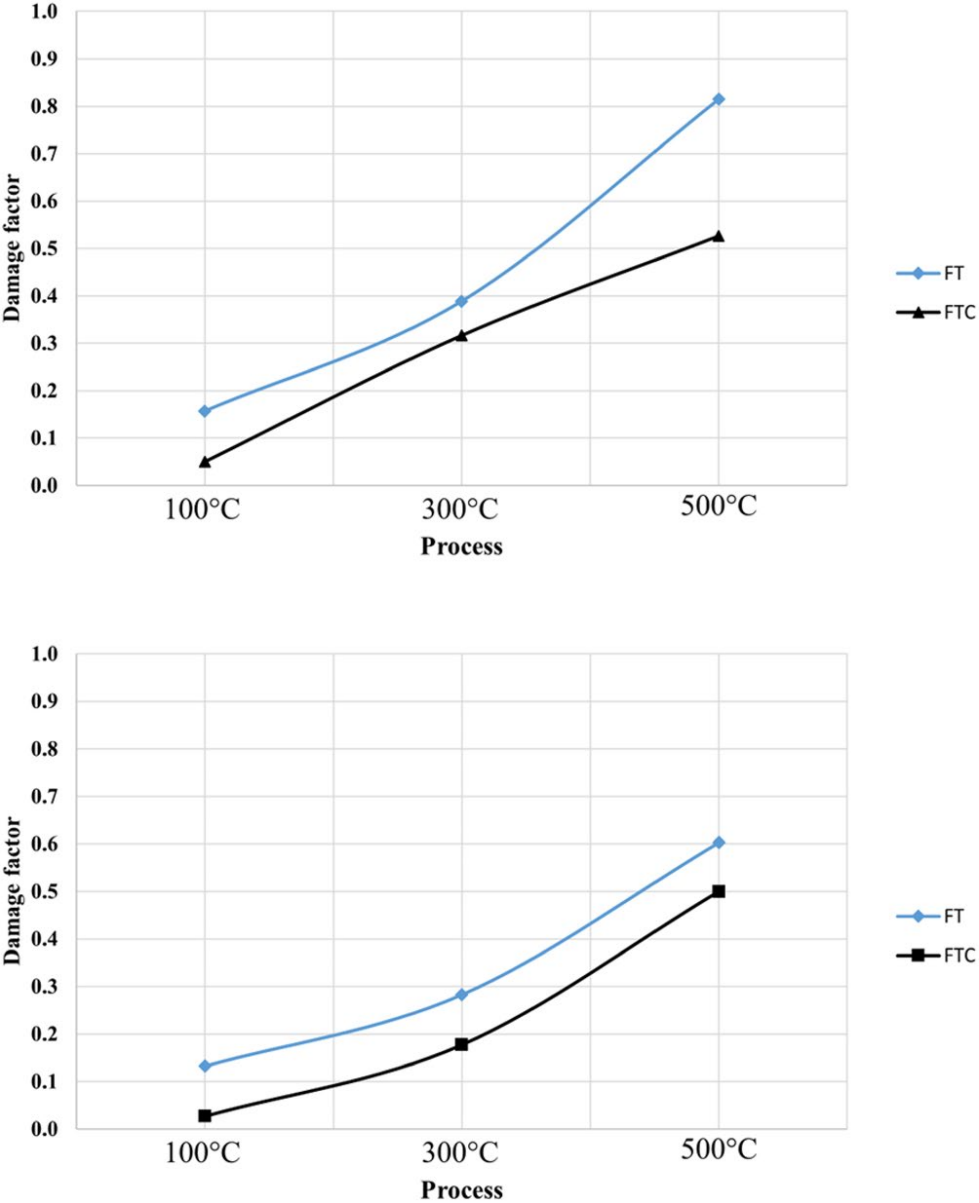
Damage factor values for FT and FTC experiments. The upper graph shows the first test, while the lower graph shows the retest.

**Figure 14:**
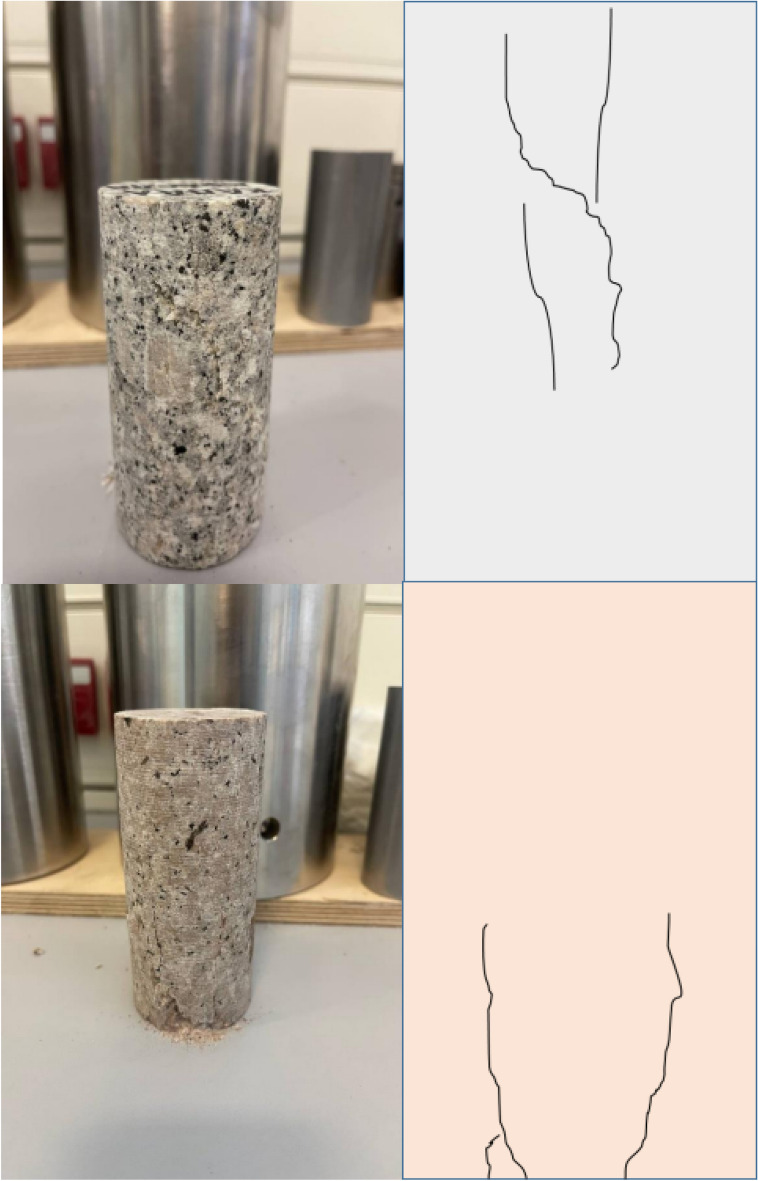
Specimens after compression tests with FT and FTC process after300 ^o^C and LN_2_ treatment.

Associated with the fracture stress of the granite, the damage factor can be estimated from the formula below^46–49^.

4 $$ {\text{D}}_{{\text{F}}} = { 1 }{-}{\text{ F}}_{{\text{c}}} /{\text{F}}_{0} $$

where D_F_ is the damage factor calculated from the fracture load, F_c_ is the maximum fracture load (kN) at different temperatures, and F_0_ is the fracture load at baseline experiment (kN). As can be noticed from Fig. 13, the D_F_ values indicate an overall upward trend with augmenting temperature and LN_2_ cooling. When the elevated temperature is smaller than 300 °C, the D_F_ value does not alter considerably. After 300 °C, the D_F_ value starts to increase significantly. At 500 °C, the D_F_ values augment further. Figure 14 shows our specimens after compression tests at 300 °C with LN_2_ treatment for FT and FTC methods.

### Brazilian tests

**Figure 15:**
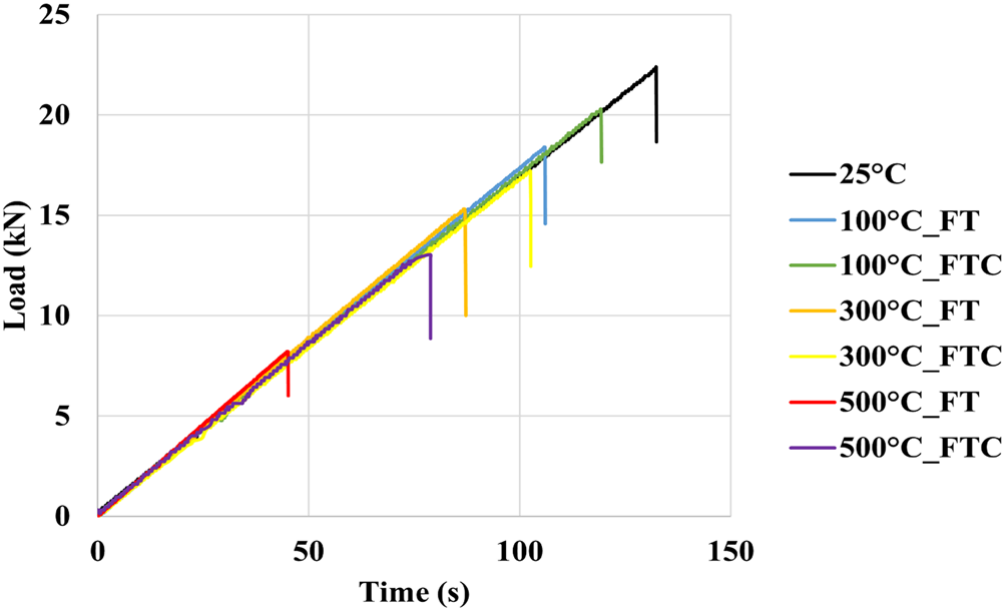
Initial load versus time for both freezing time and freezing–thawing cycle processes for Brazilian tests.

**Figure 16:**
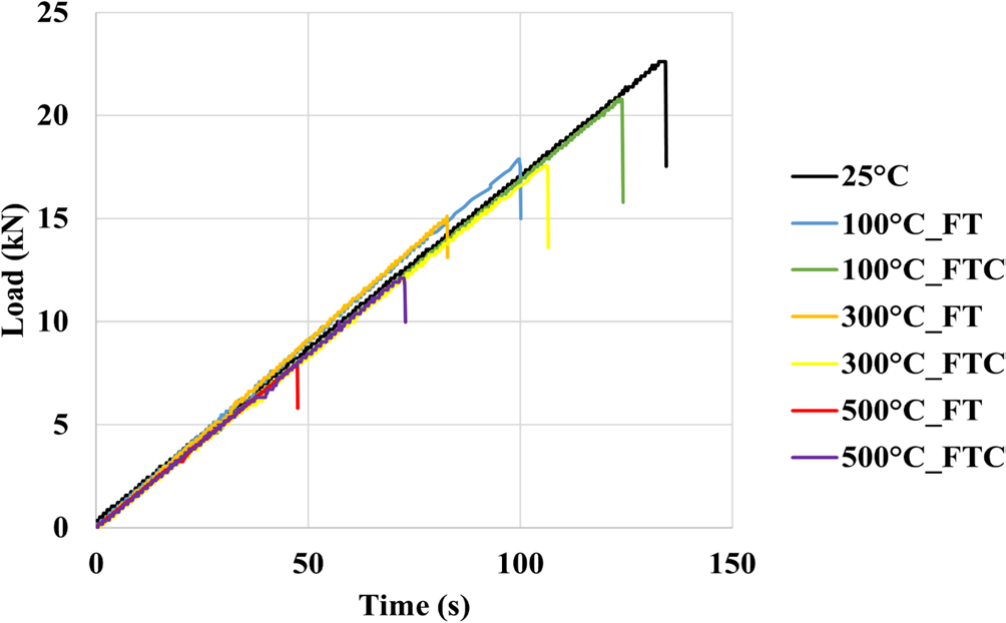
Load versus time retest for both freezing time and freezing–thawing cycle processes for Brazilian tests.

**Figure 17:**
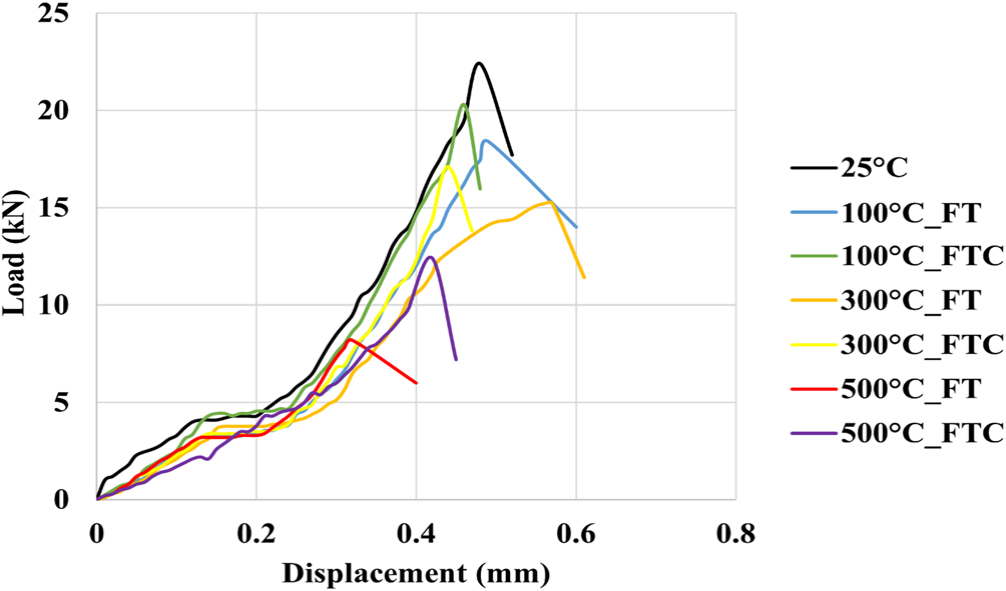
Initial load versus displacement for both freezing time and freezing–thawing cycle processes for Brazilian tests.

**Figure 18:**
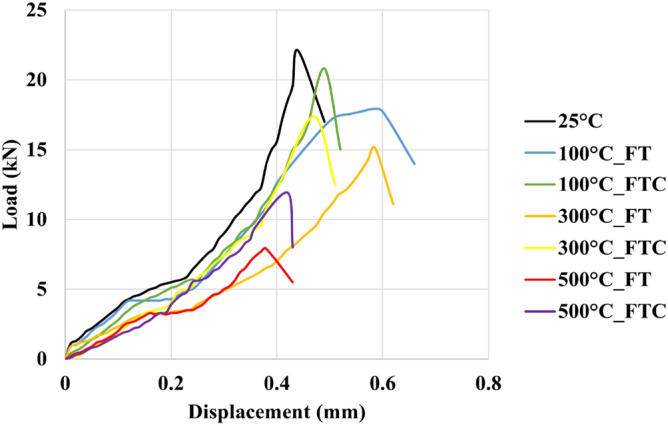
Load versus displacement retest for both freezing time and freezing–thawing cycle processes for Brazilian tests.

Brazilian compression tests were performed to examine the strength of processed granite specimens and to observe how the granite sample deformed under specific experimental conditions compared to the untreated specimens. The force and distortion results were acquired by estimating load versus time and load versus displacement for freezing and freezing–thawing cycle processes. Figures 15 and 16 present the load versus time curves for two different processes with LN_2_ treatment, and Figs. 17 and 18 present load versus displacement curves for Brazilian tests. The load reduces as the elevated temperature enhances along with LN_2_ treatment. To make the experiments (Brazilian tests) more accurate, we duplicated our experiments.

The maximum measured load in the experiment with no treatment (base sample) was 22.1 MPa (average value, Table 3). The lowest values occurred at 500 °C for both FT and FTC experiments, with 8 MPa and 12.3 MPa values, respectively. The stress of specimens with temperatures (until 300 °C in the present experimental work) after LN_2_ cooling is high with minor differences between experiments, varying from 18,5% (difference between no treatment and 100 °C with LN_2_ treatment experiments) to 64.5% (difference between no treatment and 500 °C with LN_2_ treatment experiments) for FT experiments and 5.9% to 45% for the FTC treatment process, respectively. On the other hand, the difference in maximum load between experiments for successively increasing shock temperature differences (difference between no treatment versus 100 °C and LN_2_, the difference between 100 °C and LN_2_ versus 300 °C and LN_2,_ and last, the difference between 300 °C and LN_2_ versus 500 °C and LN_2_ varies from 16.3% to 47.7% for FT experiments and from 5.9 to 30.9% for FTC experiments. The last column of Table 2 presents the standard error between repeated tests for the same conditions. The standard error for peaks in Brazilian tests differs from 0.2% for the experiment at 300 °C elevated temperature (FT) and baseline experiments (25 °C) to 1.6% for the experiment at 300 °C (FTC), confirming the consistency of our research work.

**Table 3:** Results from Brazilian test experiments for load values for both FT and FTC experiments.

Specimen no.	(°C)	Load peaks (kN), FT/FTC	Difference per experiment (%), FT/FTC	Difference per temperature (%), FT/FTC	Average value (kN), FT/FTC	Standard error (%), FT/FTC
1	25	22.2	–	–	22.1	0.2
2		22.0				
1	100	18.1/20.3	−18.5/−8.6	−18.5/−8.6	18.0/20.5	0.6/1.0
2		17.8/20.7	−19.1/−5.9	−19.1/−5.9		
1	300	15.1/17.0	−16.6/−16.3	−32.0/−23.4	15.0/17.3	0.2/1.6
2		14.9/17.5	−16.3/−15.5	−32.3/−20.5		
1	500	8.2/12.5	−45.7/−26.5	−63.1/−43.7	8.0/12.3	1.0/1.0
2		7.8/12.1	−47.7/−30.9	−64.5/−45.0		

### Fracture toughness tests

**Figure 19:**
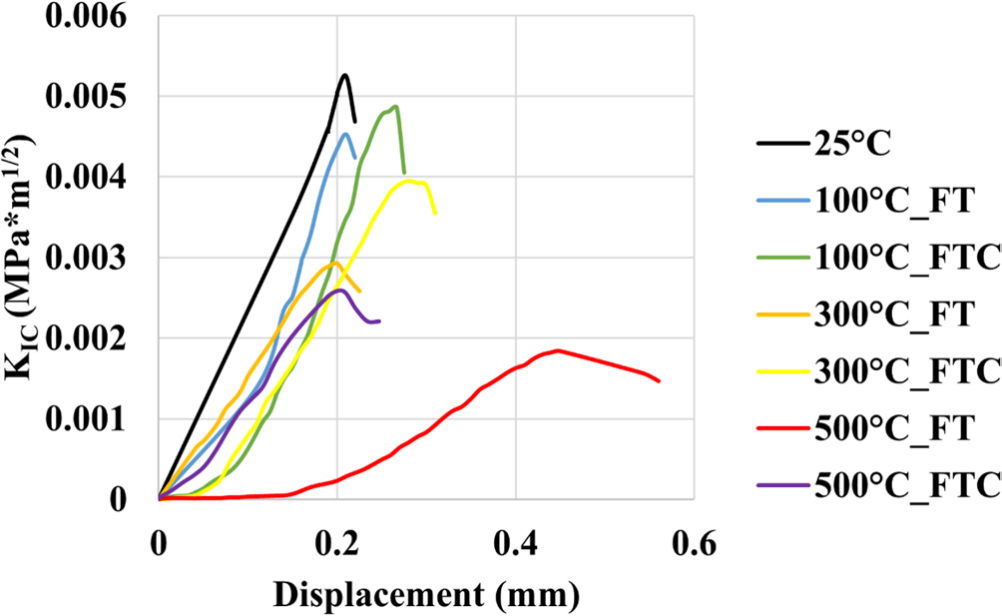
Initial fracture toughness experiments: mode I stress intensity factor versus displacement for FT and FTC experiments.

**Figure 20:**
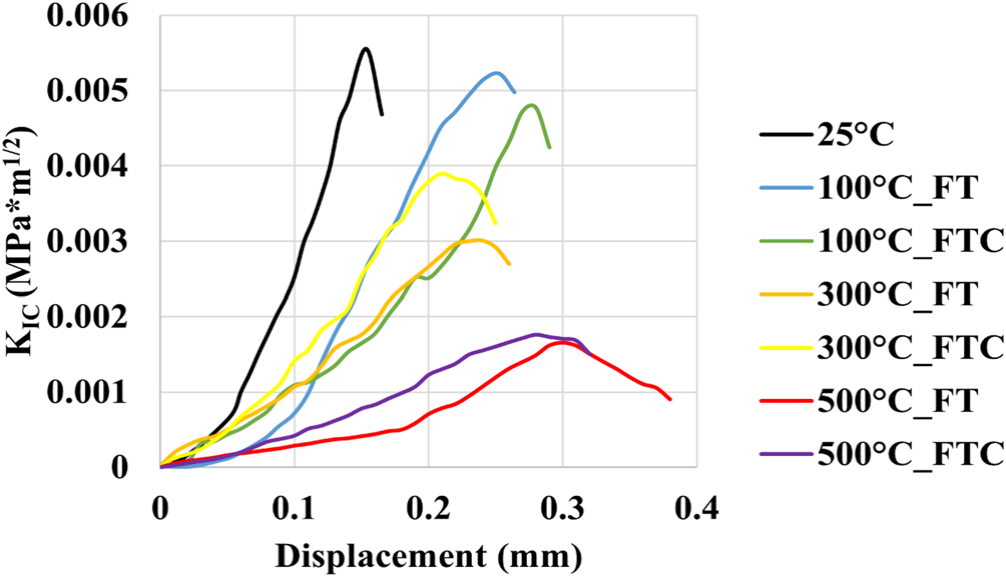
Retest fracture toughness experiments: mode I stress intensity factor versus displacement for FT and FTC experiments.

**Figure 21:**
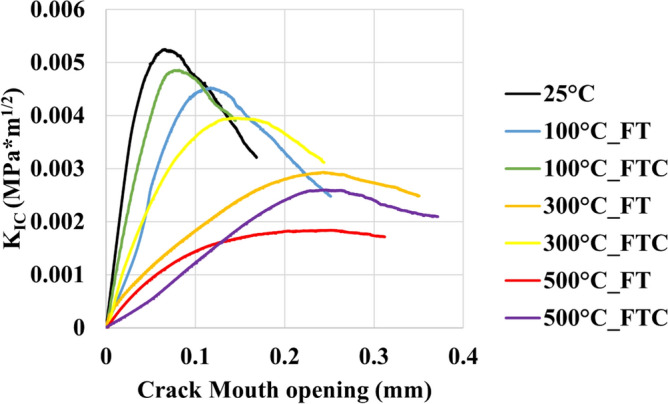
Initial fracture toughness experiments: mode I stress intensity factor versus crack mouth opening for FT and FTC experiments.

**Figure 22:**
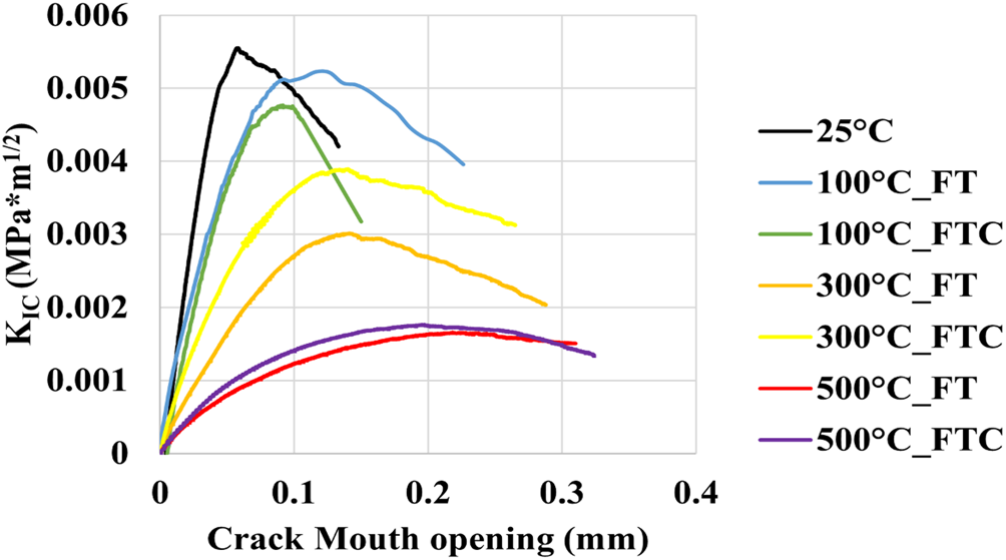
Retest fracture toughness experiments: mode I stress intensity factor versus crack mouth opening for FT and FTC experiments.

For the retested granites, the mechanical characteristics of the fracture toughness experiments are shown in Figs. 19, 20, 21 and 22, with the mode I stress intensity factor versus crack mouth opening in fracture toughness experiments presented in Figs. 19 and 20 and mode I stress intensity factor versus axial displacement in fracture toughness experiments shown in Figs. 21 and 22. The graphs show a quasi-linear enhancement of K_IC_ as a function of axial displacement and the crack mouth opening, followed by a deviation from linearity before reaching a peak (K_IC_). After the peak value, K_IC_ drops, and a big crack in the mouth opening occurs as the specimen breaks. Note that all the experiments are regarded as reliable, as the specimen failure with the formation of a crack aligned with the machined notch (i.e., mode I fracturing), as proposed by the ISRM. An optical check of the fractured surface at the end of the experimental processes indicated that the fractures initially propagated at the grain junctions and not within the grains. The average value mode I stress intensity factor was 0.0054 MPa m^1/2^. The lowest average value occurred at 500 °C with LN_2_ treatment with 0.0018 MPa m^1/2^ and 0.0024 MPa m^1/2^ for FT and FTC experiments, respectively. Furthermore, the difference per experiment (percentage difference in K_IC_ intensity factor between successive temperature increments) shows the most considerable augmentation between 300 °C and LN_2_ treatment experiments and 500 °C and LN_2_ treatment, with 42.2% and 54.1% for FT and FTC experiments, respectively. The reason is that until 300 °C and LN_2_ treatment, our samples follow an elastic behavior. In contrast, at 500 °C and LN_2_ treatment, our specimens become weaker due to increased fissures by following plastic behavior. Hence, the difference between the initial sample temperature and the boiling point of LN_2_ increases_,_ and samples fail at a lower value of K_IC_ (Figs. 19, 20, 21, 22). Table 4 shows the outcomes of fracture toughness experiments.

**Table 4:** Results from fracture toughness test values for both FT and FTC experiments.

Specimen no.	Temperature (°C)	K_IC_ peaks FT/FTC	Difference per experiment (%) FT/FTC	Difference per temperature (%) FT/FTC	Average value FT/FTC
1	25	0.0053	–	–	0.0054
2		0.0055			
1	100	0.0047/0.0049	−11.7/−9.2	−11.7/−9.2	0.0049/0.0049
2		0.0051/0.0049	−6.0/−10.7	−6.0/−10.7	
1	300	0.0029/0.0039	−37.9/−20.7	−45.2/−28.0	0.0030/0.0038
2		0.0030/0.0038	−41.0/−21.1	−44.8/−29.5	
1	500	0.0019/0.0026	−33.8/−32.5	−63.7/−51.4	0.0018/0.0022
2		0.0017/0.0018	−42.2/−54.1	−68.1/−67.6	

### Acoustic and permeability tests

**Figure 23:**
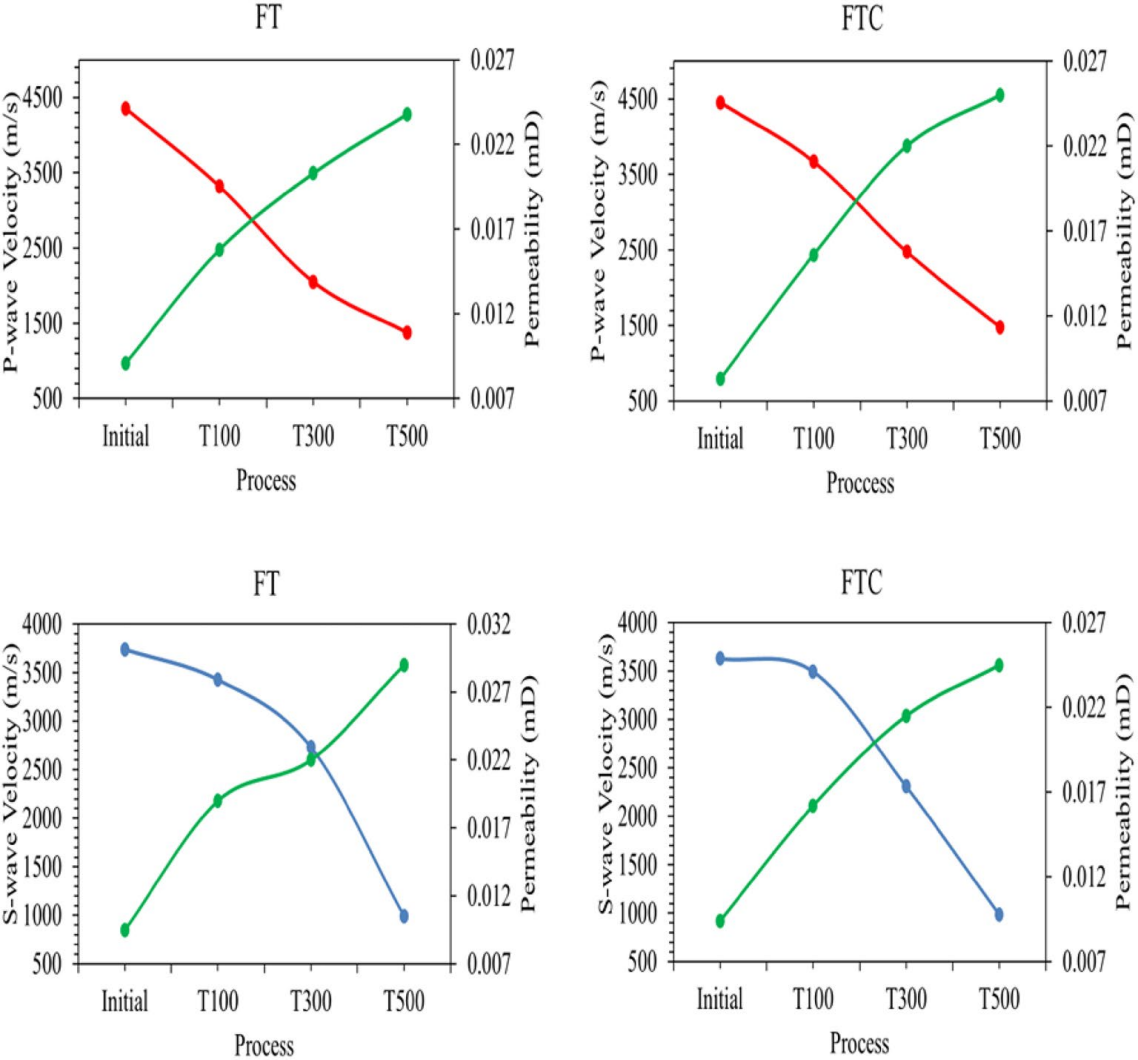
Permeability values along with p and s wave velocities.

The last estimated parameters were undestructive *p* and *s* wave velocities values along with permeabilities. The results are presented in Fig. 23. It is noticed that both v_p_ and v_s_ show a negative correlation with the heating temperature and LN_2_ treatment. With more considerable shock temperature differences, v_p_ and v_s_ both decrease. From the baseline experiment (25 °C) to 100 °C and LN_2_ treatment, the differences in v_p_ and v_s_ values were 23.8% and 8.3% for FT experiments and 17.5% and 3.7% for FTC experiments. These values increase drastically for samples equilibrated at 500 °C before LN_2_ cooling. From the baseline experiment (25 °C) to 100 °C and LN_2_ treatment, the differences in v_p_ and v_s_ values were 68.4% and 73.4% for FT experiments and 66.9% and 72.8% for FTC experiments. On the contrary, permeability values positively correlated with elevated temperature shock. From the baseline experiment (25 °C) to 100 °C and LN_2_ treatment, the value difference is 52.1% for FT experiments and 46.7% for FTC experiments. When considering 500 °C initial temperature and LN_2_ treatment, these values enhanced to 68.2% for FT and 66.8% for FTC experiments. Between the two different LN_2_ treatment processes, the permeability was enhanced more with the FT process.

## Discussion

### Effect of elevated temperature

When thermal shock occurs on the granite surface, there are changes in mechanical characteristics. As the core's initial temperature augments from 100 to 500 °C, LN_2_ exposure leads to progressively greater rock failure. Peelings were presented in specimens as the temperature difference increased. More significant thermal stresses compelled by a higher initial temperature and LN_2_ cooling facilitate the creation of micro-fissures along grain-grain boundaries, enhancing the non-uniformity and non-continuity of the granite specimen^36,38^. The thermal crack growth is an outcome of thermal stress changes due to the tensile stresses on the colder outer surface of the samples and the compressive stresses in the hotter inner portions. Moreover, micro-crevices propagate under tensile stress, come into contact with other fissures, and create complex crack networks. As the temperature shock increases, load and fracture toughness peaks are diminished. Because of thermal shock, inter-granular and intra-granular cracks are formed^46^. For no LN_2_ treatment under room environment conditions (25 °C), mineral constituents remained cemented, and no critical cracks were noticed. Since the damage of granites at 25 °C is minimal, only minor alterations in permeability can be seen. When samples are heated to 300 °C, small fissures are created. These notches were primarily placed at mineral boundaries and often did not connect (no fracture network). These crack deformations happened at grain boundaries, creating a more significant separation between minerals, indicating moderate behavior alteration^47,48^. At a temperature of 500 °C, many more fractures were formed in our specimens after LN_2_ cooling. Secondary fractures progressed, and the plastic characteristics were augmented. The dimensions of fractures were much more significant than those created below 500 °C. These fractures connected and formed “fracture networks”. The interconnected micro-fractures can compromise sample strength and increase permeability. Furthermore, it is evident that from Figs. 18 and 19, the plastic behavior of experiments with 500 °C and LN_2_ treatment for FT (mainly) and FTC processes appeared. LN_2_ exposure contributed to specimen damage since thermal stress can easily compromise the extended and weaker intergranular bonds, and thermal shock is more intense and sudden under quick cooling conditions.

### Effect of granite mineral content

Granite specimens primarily have minerals, such as quartz, feldspar, and mica, with different thermal expansion coefficients. So, there will be variations in their morphology. Makani and Vidal showed that the concentration of quartz and feldspar essentially influences granite's physical and mechanical characteristics^49^. The higher the concentration of quartz, the greater the rock's power. Conversely, the higher the concentration of feldspar, the smaller the rock power. Because of its importance, the feldspar to quartz ratio was defined as a granite characteristic, K, as

5 $$ {\text{K}} = {\text{ F}}/{\text{ Q}} $$

K is the ratio of feldspar to quartz; F is the feldspar content, comprising potash feldspar, albite, and strontium feldspar; Q is the quartz content.

Furthermore, minerals heated at higher temperatures lead to bigger disfigurements. Quartz, as a mineral, has an essential effect on heated impelled failure because of its higher thermal expansion behavior than other minerals. There were no visible fractures for experiments until 300 °C and LN_2_ treatment but just crevices. There were little cracks for experiments with 500 °C and LN_2_ treatment, with the notice that they were not deep but on the surface. Moreover, all granite specimens showed a color change from white and grey to reddish from the pre-heated temperature of 300 °C to 500 °C, but not for 100 °C, irrespective of the LN_2_ treatment. The above conclusions come by previous research works and are related to the dehydration of minerals^17,35,38,41^.

In addition, there was a difference between freezing time and freezing–thawing cycle experiments, indicating that freezing time experiments showed lower UCS peak values in both Brazilian and fracture toughness tests. While the total exposure time to LN_2_ is the same for FT and FTC processes, in FTC processes, the duration of the first thermal shock was less than in FT experiments, and the thermal gradient still existed at the end of the first cycle (5 min in LN_2_ exposure). Furthermore, successive cycles had a much-reduced thermal shock with no real impact, since samples were not returned to the initial elevated temperature before the next cycle, only given relaxation time at room conditions. This is the opposite result obtained using coal^50–53^, where frost forces were found to create fissures and crevices.

## Conclusion

More than 40 destructive (uniaxial compression, Brazilian, and fracture toughness mode I) experiments, complemented by more than 20 non-destructive (porosity and ultra-sonic) measurements, have been used to quantify better the effect of LN_2_ exposure on the strength of granite specimens. In this experimental work, we examined and compared the differences in the internal structure, mechanical characteristics, and fracture conduct of heated granites subjected to LN_2_ cooling through various mechanical experiments. Compression, Brazilian, and fracture toughness mode I test were conducted with increasing initial temperature before LN_2_ cooling (freezing time and freezing–thawing cycle processes). The main conclusions are:

- The compression tests indicated a reduction in stress as heated temperature increased along with LN_2_ treatment. Values were measured from 156.6 MPa with no treatment to 91.9 MPa for FT and 103.5 MPa for FTC experiments at 500 °C initial temperature.
- From compression tests, the Young Modulus correlated positively with increasing thermal shock, while the Poisson’s ratio correlated negatively.
- The Brazilian tests showed the load to failure of studied granite rocks in Brazilian tests progressively reduced from 22.1 MPa with no treatment to 8 MPa and 12.3 MPa for FT and FTC experiments, respectively.
- The studied granite rocks' fracture mode I intensity factor progressively decreased from 0.0054 MPa m^1/2^ with no treatment to 0.0018 MPa m^1/2^ and 0.0022 MPa m^1/2^ for FT and FTC experiments.
- In all experimental processes, experiments with an elevated initial temperature of 500 °C with subsequent LN_2_ treatment showed better outcomes with obvious new fractures or prolonged preexisting ones and creating fracture networks, which is a desired outcome. The freezing time process indicated better outcomes than the freezing–thawing cycle process.
- The last parameters for undestructive experiments of ultrasonic velocities and permeability tests indicated that permeability increased, and ultrasonic velocities (v_p_ and v_s_) decreased with increasing sample initial temperature with subsequent LN_2_ treatment.
- In all experiments, the FT process indicated better outcomes than the FTC process and followed the opposite trends compared to coal or shale experiments with LN_2_.
- Results on cryo fracturing on coal are not immediately transferrable to granite.

## Data availability 

The data that support the findings of this study are available from the corresponding author upon request.
